# Suicidality and social cognition: the association between hypomentalizing and suicide lethality

**DOI:** 10.1192/j.eurpsy.2024.398

**Published:** 2024-08-27

**Authors:** J. Andreo-Jover, K. March, E. Fernández-Jiménez, J. Fernandez Fernandez, A. Garcia Fernandez, M. P. Lopez Peña, M. Ruiz Veguilla, B. Crespo Facorro, N. Garrido Torres, A. Cebria, I. Grande, N. Roberto, W. Ayad-Ahmed, A. Pemau Gurumeta, A. Garcia Ramos, M. Diaz-Marsa, M. F. Bravo-Ortiz, A. Palao-Tarrero, V. Perez-Sola

**Affiliations:** ^1^ Universidad Autónoma de Madrid; ^2^IdiPAZ; ^3^Psychiatry, Hospital Universitario La Paz, Madrid; ^4^Universidad de Oviedo, Oviedo; ^5^Hospital Santiago de Araba, Pais Vasco; ^6^Hospital Virgen del Rocio, Sevilla; ^7^ Hospital Parc-Taulí; ^8^Hospital Clinic, Barcelona; ^9^ Universidad Complutense de Madrid; ^10^Hospital Clinico San Carlos, Madrid; ^11^Hospital Parc Salut del Mar, Barcelona, Spain

## Abstract

**Introduction:**

Suicide attempts (SA) leading to highly lethal consequences have been associated with heightened suicide planning (Barker et al., 2022), along with deficits in social cognition (Levi-Belz et al., 2022). Hypomentalizing, characterized by excessive uncertainty regarding mental states, may contribute to heightened social withdrawal and an increased risk of SA (Nestor & Sutherland, 2022). Although certain studies have identified a connection between hypomentalizing profiles and self-harm (Badoud et al., 2015), research into the lethality of SA remains limited.

**Objectives:**

This study aimed to explore the association between hypomentalizing and SA lethality.

**Methods:**

Our study encompassed a cohort of 1,371 patients who committed a SA. We conducted assessments of mentalizing using the RFQ-8 instrument, and evaluations of suicidal ideation and behavior employing the CSRSS questionnaire. Demographic and clinical characteristics were compared using the T-student and Chi-square tests. To investigate the relationship between hypomentalizing and the SA lethality, we employed logistic regression models.

**Results:**

Descriptive date are presented in Table 1. Our results show that hypomentalizing do not predict a higher SA lethality. Additionally, hypomentalizing increased the risk of SA planning (p≤0.001, B=-0.182), and SA planning predicted a higher SA lethality (see Table 2).

**Table 1.** Means Comparison for low and high lethality (N=1371)

	Low lethality N=539	High lethality N=832	p value	Effect size
Age, mean (SD)	38.65 (15.65)	41.91 (15.37)	**≤0.001**	-0.209^a^
Female sex, N (%)	392 (72.7)	571 (68.6)	0.116	0.044^b^
Educational years, mean (SD)	12.45 (2.99)	12.43 (3.41)	0.890	0.0076^a^
Employed, N (%)	220 (41.2)	332 (40)	0.692	0.012^b^
Suicide Ideation, N (%)	475 (88.1)	742 (89.2)	0.541	0.016^b^
Suicide Planning, N (%)	159 (39.2)	400 (58.1)	**≤0.001**	0.183^b^
Number of attempts, mean (SD)	3.28 (5.48)	3.63 (5.74)	0.269	-0.169^a^
RFQ, mean (SD)	4.68 (1.27)	4.56 (1.32)	0.087	0.095^a^

**Table 2.** Logistic regression analyses for high SA lethality (N=1371).

	Univariate analysis		Multivariate analysis	
	OR	p value	OR	p value
Age	1.014 (1.007-1.021)	**≤0.001**	1.014 (1.005-1.022)	**0.001**
Female sex	0.820 (0.646-1.042)	0.105		
Educational years	0.998 (0.965-1.031)	0.890		
Employed	0.952 (0.763-1.187)	0.660		
Suicide ideation	1.111 (0.790-1.562)	0.545		
Suicide planning	2.150 (1.674-2.761)	**≤0.001**	2.183 (1.697-2.808)	**≤0.001**
Number SA	1.012 (0.990-1.034)	0.277		
RFQ	0.929 (0.854-1.011)	0.088

**Conclusions:**

While the association between hypomentalizing and high SA lethality was not significant, a discernible trend toward such relationship can be noted. Further studies examining the moderating effects of planning in the association between hypomentalizing and SA lethality are required.

**Disclosure of Interest:**

None Declared

